# Generation of non-human primate CAR Tregs using artificial antigen-presenting cells, simian tropic lentiviral vectors, and antigen-specific restimulation

**DOI:** 10.1016/j.xpro.2022.101784

**Published:** 2022-11-05

**Authors:** Gavin I. Ellis, Mosha Z. Deng, Delaine W. Winn, Kimberly E. Coker, Divanshu Shukla, Vijay Bhoj, Michael C. Milone, Raimon Duran-Struuck, James L. Riley

**Affiliations:** 1 Department of Microbiology and Center for Cellular Immunotherapies, University of Pennsylvania, Philadelphia, PA 19104, USA; 2 Department of Pathobiology, University of Pennsylvania, Philadelphia, PA 19104, USA; 3 Deparment of Pathology and Laboratory Medicine, University of Pennsylvania, Philadelphia, PA 19104, USA

**Keywords:** Cell culture, Cell isolation, Flow cytometry/Mass cytometry, Immunology, Model organisms, Tissue engineering, Biotechnology and bioengineering

## Abstract

It is technically challenging to generate large doses of regulatory T cells (Tregs) engineered to express a chimeric antigen receptor (CAR) in non-human primates (NHP). Here, we have optimized the manufacturing of CAR Tregs by stringent sorting of Tregs, stimulation by artificial antigen-presenting cells, transduction by simian tropic lentiviral vectors, and antigen-specific expansion. The result of this method is highly suppressive CAR Tregs for use in a pre-clinical, large animal model of transplant tolerance.

For complete details on the use and execution of this protocol, please refer to Ellis et al. (2022).

## Highlights

• Non-human primates are ideal for pre-clinical development of CAR Treg cellular therapy
• aAPC stimulation expands NHP CAR Tregs to clinical-sized doses
• Simian tropic lentiviral vectors increase transduction relative to HIV-based vectors
• Antigen-specific restimulation enriches CAR+ Treg population *in vitro*

## Abstract (editor highlights)

Publisher’s note: Undertaking any experimental protocol requires adherence to local institutional guidelines for laboratory safety and ethics.

## Graphical abstract

**Figure:**
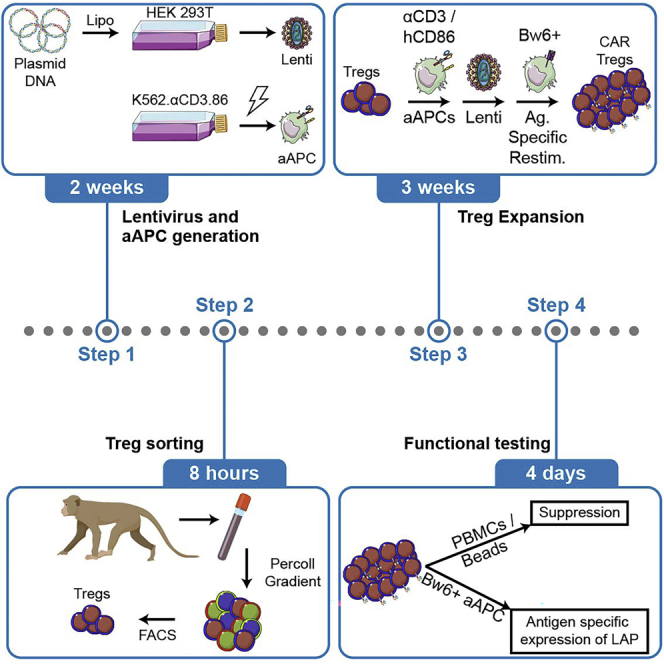

## Before you begin

The following protocol describes the expansion and transduction of CAR Tregs recognizing the NHP/human alloantigen Bw6 with CD28 and CD3ζ intracellular signaling domains. This protocol can be easily adapted to generate CAR Tregs with other specificities or to generate effector CAR T cells. Here, we describe the protocol for *Cynomolgus macaque*, but we have also generated CAR T cells from *Rhesus macaque* with equal success. Before starting, generate aliquots of simian tropic χHIV lentiviral vectors, irradiated K562 artificial antigen presenting cells (aAPCs), and NHP α-CD3/α-CD28 beads to have on hand.

### Institutional permissions

Obtain institutional permission to perform animal studies and collect peripheral blood from NHPs under an approved Institutional Animal Care and Use Committee (IACUC) or Institutional Review Board protocol. Our protocol was approved by the University of Pennsylvania Institutional Animal Care and Use Committee (IACUC).

#### Manufacturing of irradiated K562 aAPCs for T cell stimulation

**Timing: 2 weeks**

The following method will generate large quantities of irradiated K562 aAPCs for stimulating and expanding NHP T cells. The aAPCs used here were generated by transducing K562 cells with both α-CD3 CAR and human CD86 lentiviral vectors (K562.F12Q.86) or with HLA-B7 (Bw6+) and human CD86 vectors (K562.Bw6.86), performing single cell sorting of double positive clones, and characterizing the stability of transgene expression over time before selecting an optimal clone (Ellis et al., 2022). In our hands, K562-based aAPC stimulation results in larger T cell numbers versus bead-based stimulation (Ellis et al., 2022; Hippen et al., 2008, 2011; Maus et al., 2002; Suhoski et al., 2007; Thomas et al., 2002; Ye et al., 2011). We have used these cells successfully to stimulate *Cynomolgus macaque* (Ellis et al., 2022) and *Rhesus macaque* (Rust et al., 2020) T cells.

1. Begin expansion of K562.F12Q.86 and K562.Bw6.86 cells.
  a. Thaw one vial of cryopreserved K562 cells rapidly in a 37°C water bath.
  b. Add cells to a 15 mL conical tube.
  c. Add room temperature or warmer R10 medium dropwise, shaking between every few drops.
  d. Spin down cells at 485 g for 5 min to wash.
    ***Note:*** All centrifugation steps should be performed at room temperature.
  e. Resuspend cells in R10 medium and count.
  f. Adjust cells to 200k cells/mL and incubate overnight in an appropriately sized cell culture flask in a 37°C incubator with 5% CO_2_.
    ***Note:*** As K562 cells are grown in suspension, the size of the flask is dependent on number of cells, and is selected to ensure efficient gas exchange by plating at 0.2–0.5 mL/cm^2^ ratio of volume to flask surface area.
2. The following day, passage cells for further propagation in a cell culture flask.

***Note:*** Cells should be diluted with R10 medium to 50k cells/mL every other day or 25k cells/mL every 3 days to maintain concentration under 400k cells/mL.

3. After 1 week of passaging, add 1.25 × 10^6^ K562s to each well of a G-Rex 6 well plate.
4. Fill each well up to 100 mL with fresh R10 medium and return plate to incubator.
5. Grow cells undisturbed for 1 week.
6. After 1 week, remove medium in each well so that ∼5 mL remains.
7. Combine cells from all wells and bring up to 50 mL with R10.
8. Put cells in T150 flask and deliver 100 gy. of irradiation.
9. Wash cells with R10 medium and resuspend cells for cryopreservation at a concentration of 2.5–10 × 10^6^ cells/mL in 90% FBS + 10% DMSO.
10. Pipette cells into cryovials, then put cryovials in room temperature freezing container for freezing at −80°C overnight.
11. The following day, move cryovials to liquid nitrogen for long term storage.

#### Generation of χHIV lentiviral vectors

**Timing: 4 days**

**Figure 1:**
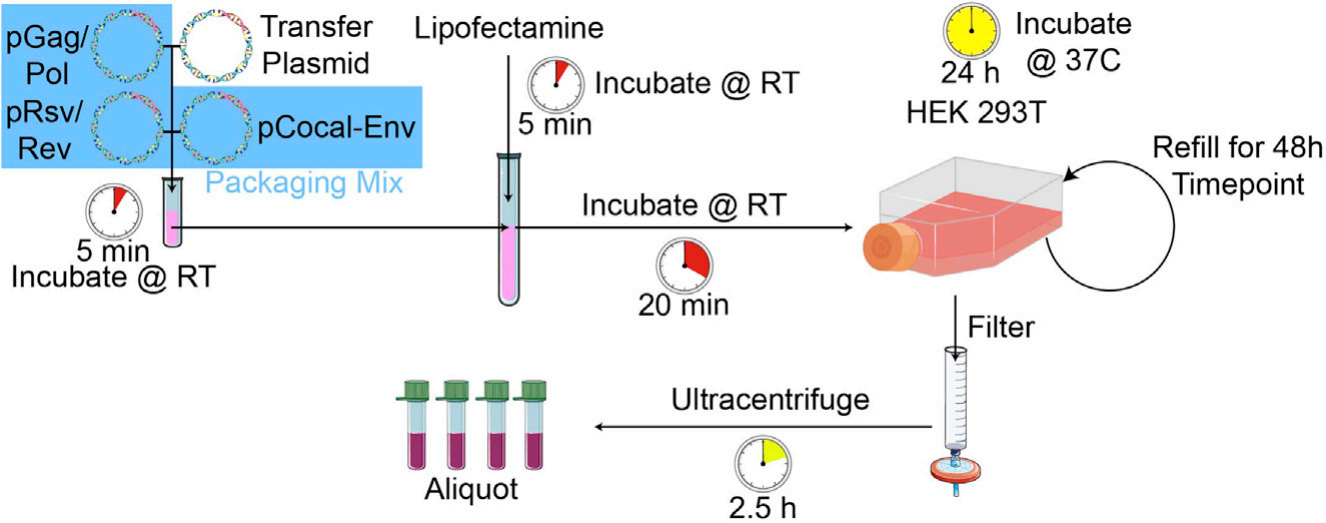
Outline of simian tropic lentiviral vector generation

**Figure 2:**
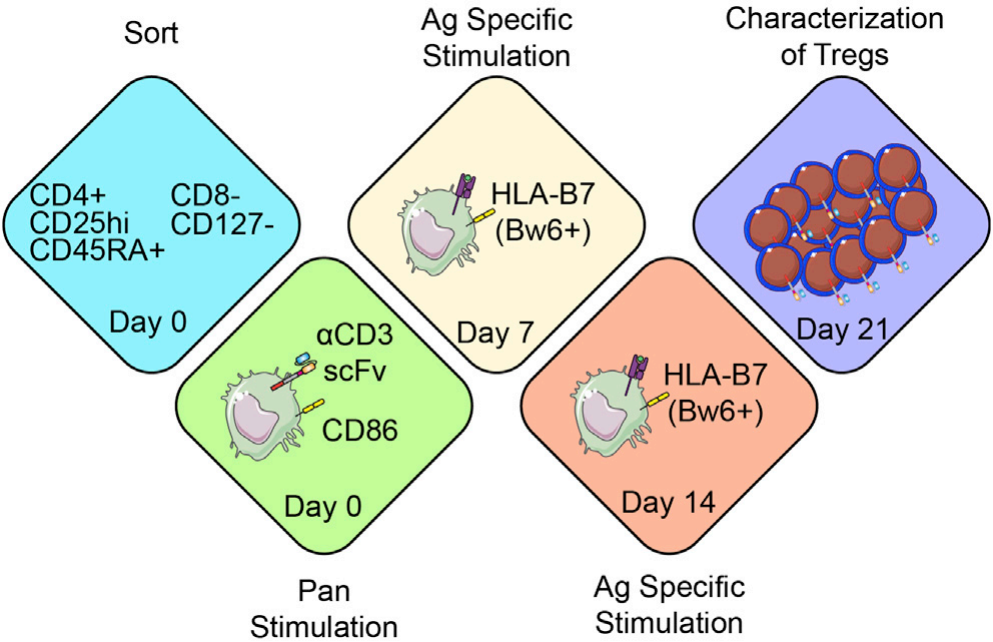
Outline of CAR Treg manufacturing

NHP T cells express restriction factors that can reduce transduction efficiency by HIV based lentiviral vectors (Ellis et al., 2022; Hatziioannou et al., 2006; Richardson et al., 2014; Stremlau et al., 2004). Therefore, our simian tropic lentiviral packaging mix contains an HIV-SIV chimeric gag-pol (χHIV) (Hatziioannou et al., 2006; Uchida et al., 2009) and are pseudotyped with Cocal virus envelope glycoprotein (Trobridge et al., 2010). For best results, all DNA should be endotoxin free and CAR transgene should be placed under the EF1α promoter in the transfer plasmid (Leibman et al., 2017; Milone et al., 2009). See Figure 1 and Figure 2 for protocol outline and summary.

12. Aliquot lentiviral plasmid packaging mix.
  a. Combine 3 μg Cocal-Env plasmid, 18 μg HIV_REV_ expression plasmid, and 18 μg HIV-SIV chimeric gag-pol plasmid.
  b. Fill up to 40 μL with nuclease free water.

***Note:*** Aliquots can be stored at −20°C to −80°C for >2 years.

13. Generate transfer plasmid DNA of choice (Bw6-28z CAR is used in this protocol as described in (Ellis et al., 2022)) with endotoxin free maxiprep kit.
14. 24 h before lipofection, seed 8–10 × 10^6^ HEK 293T cells in a T150 tissue culture flask in 30 mL R10 medium so that they are 70%–90% confluent the following day at the time of lipofection.
  ***Note:*** HEK 293T cells detach from the plate very easily. Add/remove media to the top side of the flask.
  a. Remove medium from HEK 293T cells.
  b. Wash cells twice gently with 10 mL PBS.
  c. Add 3 mL trypsin to the flask and incubate for 30 s at room temperature.
  d. Add 7 mL R10 to the flask to wash cells off bottom of the flask.
  e. Add cells to a 50 mL conical tube.
  f. Wash bottom of the flask with 10 mL R10 twice more and add to 50 mL conical tube.
  g. Centrifuge cells for 5 min at 485 g at room temperature and resuspend in 10 mL R10.
  h. Count cells.
  i. Resuspend 8–10 × 10^6^ cells in 30 mL R10 and culture them in a separate T150 tissue culture flask for each virus to be made.
  j. Incubate flasks for 24 h.
15. On the day of lipofection, use a microscope to check that the HEK 293T cells are 70%–90% confluent and equally distributed across the bottom of the flask.
16. Lipofect HEK 293T cells with viral DNA.
  a. Fill a 15 mL conical tube and a sterile FACS tube with 2.3 mL each of Opti-MEM.
  b. Transfer 27 μg of transfer plasmid to 1 vial of thawed lentiviral plasmid packaging mix generated in step 12.
  c. Transfer all DNA to FACS tube containing Opti-MEM.
  d. Add 87 μL Lipofectamine 2000 to the 15 mL tube containing Opti-MEM.
  e. Incubate both tubes for 5 min at room temperature.
  f. Add contents of FACS tube to 15 mL tube dropwise.
  g. Incubate for 20 min at room temperature.
  h. Remove media from HEK 293T cells.
  i. Add lipofectamine/DNA mixture directly to the HEK 293T cells gently.
  j. Let sit for 30 s at room temperature.
    ***Note:*** HEK 293T cells detach from the plate very easily. Add/remove media on the top side of the flask and add lipofection solution very gently directly on top of the cells.
  k. Add 30 mL R10 to the flask and return to 37°C incubator.
17. 24 h later, harvest viral supernatant.
  a. Collect the viral supernatant into a 50 mL conical tube.
  b. Replace supernatant with 36 mL R10 and put back in incubator for 48 h viral harvest.
  c. Spin 24 h viral supernatant for 5 min at 485 g at room temperature.
  d. Spray ultracentrifuge tubes with 70% ethanol.
  e. Wash out ethanol with sterile PBS and place inside ultracentrifuge rotor bucket.
  f. Remove plunger from 60 mL syringe.
  g. Attach 45 μm filter to syringe with Leur lock.
  h. Carefully pour viral supernatant into syringe.
  i. Filter supernatant into ultracentrifuge tube inside rotor bucket.
  j. Spin cells at 114,000 g at 4°C for 2.5 h with slow deceleration.
    ***Note:*** Lentiviral vectors are heat labile. Following ultracentrifugation, keep rotor buckets on ice.
  k. Pull ultracentrifuge tubes from rotor buckets with tweezers.
  l. Aspirate medium until ∼400 μL remains.
  m. Resuspend viral pellet in residual 400 μL gently to avoid making bubbles.
  n. Add ∼100 μL virus to each of 4 cryotubes and freeze quickly on dry ice.
  o. When supernatant is solid, move cryovials to −80°C freezer for long term storage.
18. Repeat step 17 for 48 h viral harvest.

#### Loading of T cell stimulation particles with α-CD3 and α-CD28 for Treg suppression assay

**Timing: 2 h**

19. Pipette 100 μL of CD3-Biotin and 100 μL of CD28-Biotin into a sterile Eppendorf tube and mix well.
20. Vortex anti-Biotin MACSiBead particles and add 100 × 10^6^ beads (500 μL) to the antibody-containing Eppendorf tube.
21. Add 300 μL flow buffer to bring total volume to 1 mL.
22. Incubate bead/antibody mix for 2 h at 4°C while rocking.
23. Store beads at 4°C for up to 4 months.

## Key resources table

**Table:** 

REAGENT or RESOURCE	SOURCE	IDENTIFIER
**Antibodies**		

CD4-BV421 (1:200 dilution)	BioLegend	Cat#317434; Clone: OKT4; RRID: AB_2562134
CD4-AF488 (1:100 dilution)	BioLegend	Cat#317420; Clone: OKT4; RRID: AB_571939
CD4-BV605 (1:100 dilution)	BioLegend	Cat#317438; Clone: OKT4; RRID: AB_11218995
CD8-BV510 (1:100 dilution)	BioLegend	Cat#301048; Clone: RPA-T8; RRID: AB_2561942
CD25-BV421 (1:100 dilution for extracellular only staining, 1:20 dilution if performing intracellular staining)	BioLegend	Cat#302630; Clone: BC96; RRID: AB_11126749
CD127-PE (1:100 dilution)	BioLegend	Cat#351301; Clone: A019D5; RRID: AB_10720815
CD45RA-FITC (1:100 dilution)	BD Biosciences	Cat#556626; Clone: 5H9; RRID: AB_396498
Fixable Viability Dye eFluor 780 (1:2000 dilution)	eBioscience	Cat#65-0865-18
CTLA-4-BV786 (1:100 dilution)	BD Biosciences	Cat#563931; Clone: BNI3; RRID: AB_2738491
Helios-PE/Cy7 (1:100 dilution)	BioLegend	Cat#137236; Clone: 22F6; RRID: AB_2565990
FoxP3-PE/Dazzle 594 (1:20 dilution)	BioLegend	Cat#320126; Clone: 206D; RRID: AB_2564025
HLA-B∗07:02 HIV nef TPGPGVRYPL-PE (1:200 dilution)	MBL International	Cat#TS-M054-1
LAP-APC (1:50 dilution)	BioLegend	Cat#349706; Clone: TW5-6H10; RRID: AB_10680787
CD25-PE/Dazzle 594 (1:100 dilution for extracellular only staining, 1:20 dilution if performing intracellular staining)	BioLegend	Cat#302646; Clone: BC96; RRID: AB_2734260
Cynomolgus CD3 epsilon protein (1:100 dilution)	ACROBiosystems	Cat#CDE-C5526
His Tag-AF647 (1:100 dilution)	BioLegend	Cat#652513; Clone: J099B12; RRID: AB_2716153
CD86-BV421 (1:100 dilution)	BioLegend	Cat#374212; Clone: BU63; RRID: AB_2728394

**Biological samples**		

Fetal Bovine Serum	Avantor Seradigm	Cat#97068-085

**Chemicals, peptides, and recombinant proteins**		

Penicillin-Streptomycin	Gibco	Cat#15140122
Proleukin (aldesleukin) IL-2	Clinigen	N/A
Percoll	MilliporeSigma	Cat#P4937
ACK Lysing Buffer	Quality Biological	Cat#118-156-101
GlutaMAX Supplement	Gibco	Cat#35050061
1 M HEPES	Gibco	Cat#15630080
RPMI 1640	Gibco	Cat#11875085
Opti-MEM	Gibco	Cat#31985070
Lipofectamine 2000	Invitrogen	Cat#11668027
DMSO	Sigma-Aldrich	Cat#D2650
Trypsin-EDTA (0.05%), phenol red	Gibco	Cat#25300-054

**Critical commercial assays**		

True-Nuclear Transcription Factor Buffer Set	BioLegend	Cat#424401
T Cell Activation/Expansion Kit, non-human primate	Miltenyi Biotec	Cat#130-092-919
CellTrace Violet	Molecular Probes	Cat#C34557
PureLink Expi Endotoxin-Free Maxi Plasmid Purification Kit	Invitrogen	Cat#A31217

**Experimental models: Cell lines**		

K562	ATCC	Cat#CCL-243
HEK 293T	ATCC	Cat#CRL-3216

**Recombinant DNA**		

HIV-SIV chimeric gag-pol plasmid	Dr. Vijay Bhoj	N/A
Cocal-Env plasmid	Synthesized and codon optimized by ATUM, Newark, California	N/A
HIV_REV_ expression plasmid	Synthesized by DNA 2.0	N/A
Bw6-28z transfer plasmid	Synthesized by DNA 2.0, designed and cloned as described in (Ellis et al., 2022)	N/A

**Software and algorithms**		

FlowJo	BD	https://www.flowjo.com

**Other**		

X-Rad320ix irradiator	Precision X-Ray	N/A
G-Rex 6M well plate	Wilson Wolf	Cat#80660M
Ultra-Clear ultracentrifuge tubes	Beckman Coulter	Cat#344058
Optima XPN-100 ultracentrifuge with SW-32 Ti swinging bucket rotor	Beckman Coulter	N/A
0.45 μm syringe filter	Pall	Cat#4654
50 mL syringe	BD	Cat#309653
FACS Jazz	BD	N/A
96 well flat bottom plate	Corning	Cat#3596
96 well round bottom plate	Corning	Cat#3799
70 μm cell strainer	Corning	Cat#08-771-2
Polystyrene FACS tube with cell strainer snap cap	Falcon	Cat#352235
Polypropylene FACS tube with cap	Falcon	Cat#352063
Corning CoolCell LX freezing container	Corning	Cat#07-210-001

## Materials and equipment

**Table:** 60% Percoll solution

Reagent	Final concentration	Amount
Filtered Milli-Q water	N/A	150 mL
10× PBS	1×	50 mL
Percoll	60% (v/v)	300 mL

Keep sterile and store at room temperature for 1 year.

**Table:** R10 medium

Reagent	Final concentration	Amount
RPMI-1640 with glutamine	N/A	870 mL
Heat inactivated FBS	10%	100 mL
10× Glutamax	1×	10 mL
10× Penicillin / Streptomycin	1×	10 mL
1 M HEPES	10 mM	10 mL

Keep sterile and store at 4°C for up to 1 month.

**Table:** Flow buffer

Reagent	Final concentration	Amount
PBS without Ca2+ or Mg2+	N/A	978 mL
Heat inactivated FBS	2%	20 mL
0.5 M EDTA	1 mM	2 mL

Keep sterile and store at 4°C for up to 2 months.

## Step-by-step method details

### Isolation of PBMCs from *Cynomolgus macaque* whole blood

**Timing: 3 h**

The isolation of PBMCs from *Cynomolgus macaque* blood is not as efficient as that of human or even *Rhesus macaque*. We have found that Percoll gives the best results. The *Cynomolgus* PBMC layer will contain many more red blood cells than human, so ACK lysis is essential for efficient cell sorting.

1. Dispense anti-coagulated blood into a 50 mL conical tube and spin at 650 g for 10 min at room temperature with the deceleration and acceleration on lowest speed to avoid disruption of plasma layer atop the RBC/PBMC layer.
2. Pipette off the clear plasma (upper layer) leaving behind RBC/PBMC enriched layer.
3. Dilute remaining RBC/PBMC layer with 1 volume of PBS.
4. Place 15 mL room temperature 60% Percoll solution into a 50 mL conical, making sure to avoid getting Percoll on the sides of the tube.
5. Slowly, overlay up to 35 mL blood on top of the Percoll solution.
6. Centrifuge blood for 30 min at 650 g with the deceleration and acceleration on lowest speed.
7. Pipette off the top layer until ∼2.5 cm above interphase.
8. Use a pipette to collect the hazy middle layer containing PBMCs and put into a new tube.
9. Fill up new 50 mL conical tube with PBS.
10. Wash cells by spinning at 650 g for 10 min with the deceleration and acceleration returned to their highest settings.
11. Remove supernatant and lyse red blood cells.
  a. Resuspend cell pellet in 1 mL of ACK lysis buffer.
  b. Fill up tube to 50 mL with ACK lysis buffer.
  c. Rock tube gently for 8 min at room temperature.
  d. Spin cells for 10 min at 650 g.

***Note:*** Do not exceed 8 min of lysing time for optimal lymphocyte recovery. If cellular debris is visible following lysis, strain sample through a 70 μm filter into a new 50 mL conical tube before centrifugation.

12. Resuspend cells in 300 μL of flow cytometry buffer and move to a sterile FACS tube.

### Sorting of Tregs from PBMCs

**Timing: 5 h**

**Figure 3:**
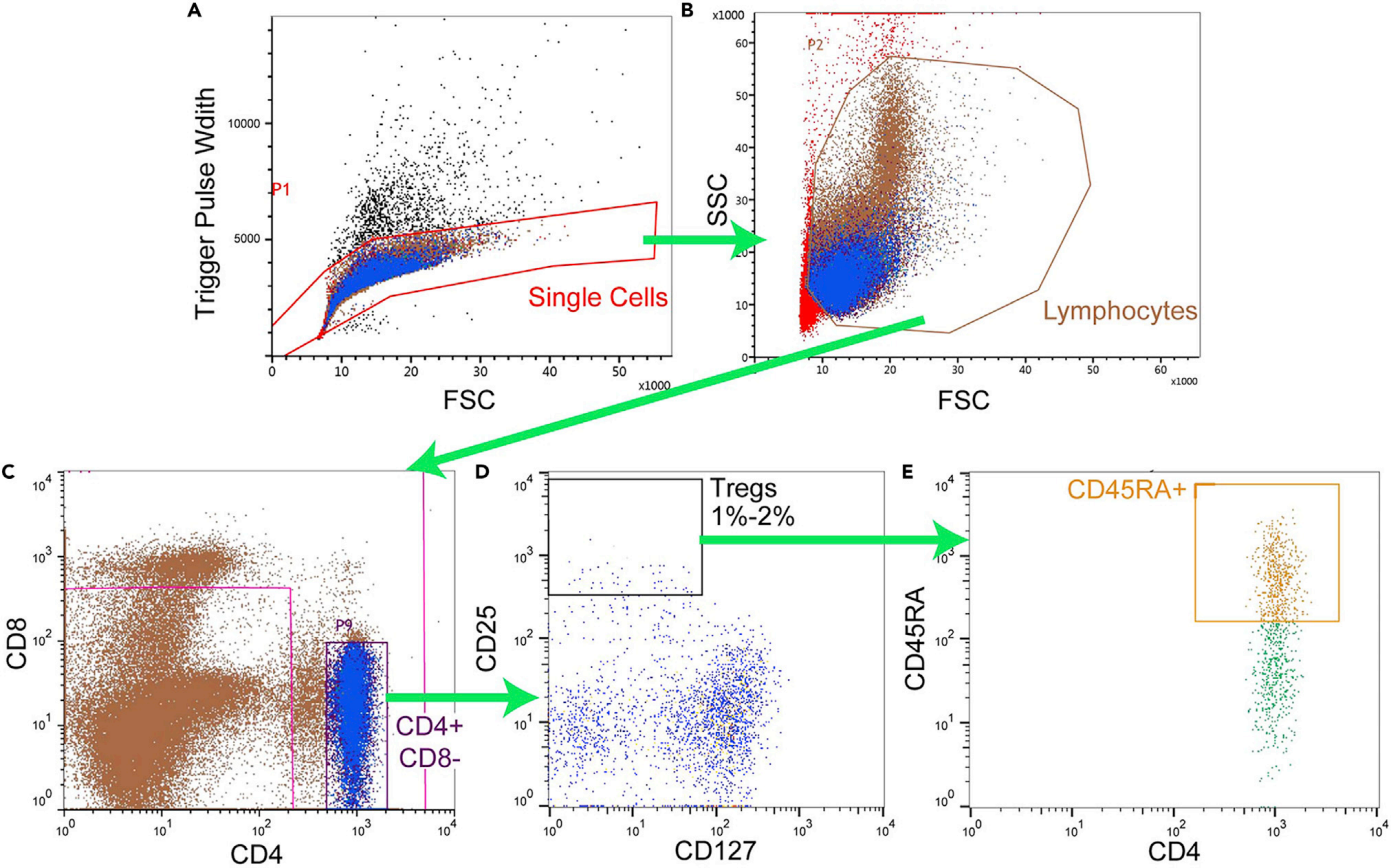
Hierarchical gating strategy for NHP Treg sorting (A) Trigger pulse width vs. FSC gates on single cells. (B) SSC vs. FSC gates on lymphocytes. (C) CD4+ CD8- gating removes both CD4- CD8+ and CD4+ CD8+ cells. (D) Among CD4+ CD8- cells, gate on the top 1%–2% of CD25+ CD127- cells. (E) finally, gate on CD45RA+ cells.

To minimize cell death, we perform a low-pressure sort using a 100 μm nozzle into 5 mL polypropylene tubes containing 1 mL of R10 medium as a cushion. Platelets will outnumber lymphocytes in the sample to be sorted. Use your flow sorter’s FSC threshold to eliminate platelets from the screen. Though many platelets will remain in the Treg population following sorting, we have not seen this impact any downstream applications. Figure 3 depicts the gating strategy we use to isolate Tregs in *Cynomolgus macaque*.

13. Make antibody staining master mix containing titrated amounts of the following antibodies:
  a. CD4-BV421 (OKT4).
  b. CD8α-BV510 (RPA-T8).
  c. CD25-PE/Dazzle 594 (BC96).
  d. CD127-PE (A019D5).
  e. CD45RA-FITC (5H9).

***Note:*** The concentration of antibodies used for flow cytometry throughout this protocol will need to be titered by each individual lab to ensure optimal performance on their flow sorter. We stain up to 100 million cells in 600 μL of flow buffer.

14. Add antibody staining master mix to cells.
15. Vortex tubes to mix well.
16. Put tubes in 4°C refrigerator for 15 min.
17. Wash cells with 3 mL flow buffer, spin for 10 min at 650 g at room temperature.
18. Resuspend cells in 500 μL flow buffer.
19. Filter cells through 35 μM strainer top moved from a polystyrene FACS tube onto a sterile polypropylene FACS tube.
20. Count cells and adjust to 20 × 10^6^ cells/mL with flow buffer.
21. Sort Tregs using the following successive gates (Figures 3A–3E):
  a. Trigger Pulse Width vs. FSC-A for single cells.
  b. SSC-A vs. FSC-A to gate on lymphocytes.
  c. CD4+ CD8-.
  d. CD25+ CD127- (take top 1%–2%).
  e. CD4+ vs. CD45RA+.

### Expansion and transduction of CAR Tregs

**Timing: 3 weeks**

**Figure 4:**
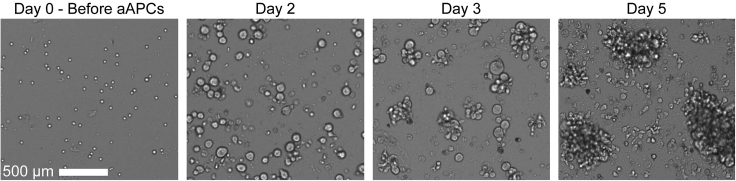
Example growth of NHP CAR Tregs over time

**Figure 5:**
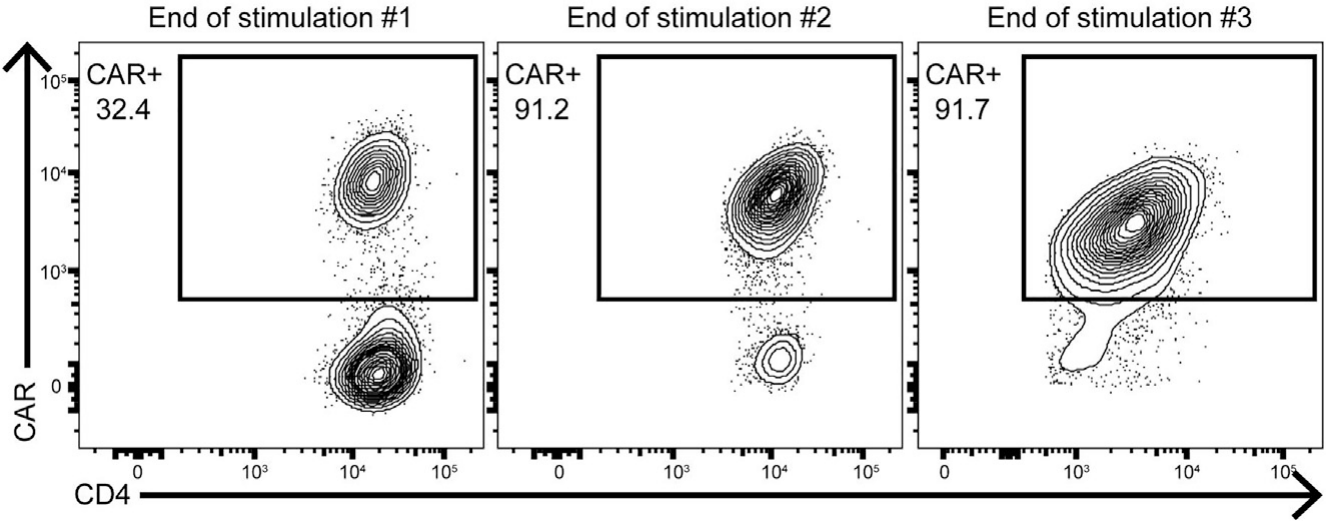
Enrichment of CAR+ Tregs during manufacturing CAR staining at the end of each week of Treg growth demonstrating enrichment of CAR+ cells in the Treg product.

**Figure 6:**
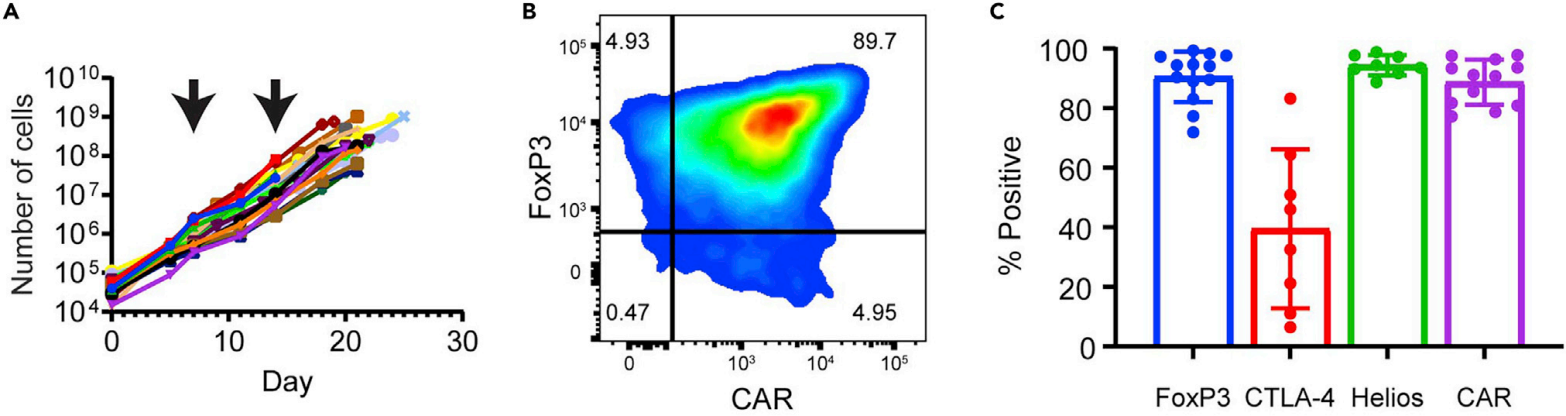
CAR Treg growth and flow phenotype (A–C) Expected CAR Treg growth (A) and expression of FoxP3, CTLA-4, Helios, and CAR on final CAR Treg product (B and C). Each line in panel A represents a single expansion of Tregs, and each arrow represents the day of restimulation with irradiated K562.Bw6.86 cells. Panels B and C represent data from day 18–21 of expansion.

The goal of this step is to take stringently sorted Tregs and expand them into clinical sized doses of CAR Tregs. As few as 25k Tregs is sufficient to grow clinical sized doses of CAR Tregs. On each day of (re)stimulation, it is critical that aAPCs make physical contact with the T cells, so err on the side of choosing a plate or flask with a smaller surface area rather than a larger one. Since cells transduced with functional levels of CAR are exclusively re-stimulated by K562.Bw6.86 cells, absolute growth can be minimal between weeks 1–2 despite an increase in the percentage of CAR+ Tregs. This contrasts with antigen non-specific stimulation during week 1, where each cell is stimulated. Microscopy of stimulated Tregs can be seen in Figure 4. Figure 5 demonstrates the enrichment of CAR+ cells by antigen specific restimulation of CAR Tregs, while Figure 6 depicts the expected growth of Tregs along with expected expression of FoxP3, CAR, CTLA-4, and Helios.

22. Spin down sorted Tregs and count cells.
23. Plate 25k–100k Tregs in 200 μL R10 medium with 100k irradiated K562.F12Q.86 cells and 300 IU/mL IL-2 in 96 well flat bottom plate.

***Note:*** We also expand CD4+ CD25- effector T cells (Teffs) to be used as FoxP3- flow and suppressor assay controls with this same protocol with 100 IU/mL of IL-2 instead of 300 IU/mL.

24. After 48 h, add ½ vial of Bw6-28z virus dropwise, making sure not to disturb any clumps.
25. 24 h later, move cells to 48 well plate.
26. Double the volume of R10 and add IL-2 back to 300 IU/mL, assuming consumption. Place cells back in incubator.
27. On day 5, count cells and adjust to 500k cells/mL with R10.
28. Add IL-2 to cells at 300 IU/mL, assuming consumption.
29. On day 7, count cells and take 50k cells for surface staining flow cytometry to assess viral transduction percentage, staining in 100 μL of flow buffer containing titrated amounts of the following:
  a. Fixable Viability Dye eFluor 780.
  b. CD4-BV421 (OKT4).
  c. CD8α-BV510 (RPA-T8).
  d. CD25-PE/Dazzle 594 (BC96).
  e. HLA-B7 Tetramer-PE.
30. Take remaining cells and adjust to 500k cells/mL with R10 medium.
31. Add 1 irradiated K562.Bw6.86 cell per Treg and add 300 IU/mL IL-2, assuming consumption.

***Note:*** Any irradiated target antigen+ cells can be used for restimulation. In this example, Bw6+ PBMCs (30 gy. irradiated) is an alternative.

32. On day 9, double media with R10 containing 600 IU/mL IL-2 and move cells to appropriate plate or flask.
33. On day 11, count cells and adjust to 500k cells/mL with R10 medium.
34. Add IL-2 to 300 IU/mL, assuming consumption.
35. On day 14 / 16 / 18, repeat the steps from days 7/ 9 / 11, but with a 1:2 ratio of aAPCs to Tregs.
36. When cells reach resting state, usually around 375–400 fL in volume as seen on a Coulter counter during cell counting, freeze up to 50 × 10^6^ Tregs in 1 mL of 90% FBS + 10% DMSO solution.
37. To assess Treg product by flow cytometry, first wash 500k cells in 3 mL flow buffer and then resuspend cells in 100 μL flow buffer containing titrated amounts of the following:
  a. CD4-BV605 (OKT4).
  b. CD8α-BV510 (RPA-T8).
  c. CD127-PerCP/Cy5.5 (A019D5).
  d. CD25-BV421 (BC96).
  e. Fixable Viability Dye eFluor 780.
38. Incubate in 4°C refrigerator for 30 min.
39. Wash cells with 3 mL flow buffer.
40. Spin down for 5 min at 485 g.
41. During spin, add 250 μL 4× TrueNuclear Fix Concentrate to 750 μL Fix Diluent to make 1× Fix Solution.
42. Decant liquid and resuspend cells in 1 mL TrueNuclear Fix Solution.
43. Vortex cells well and incubate at room temperature for 30 min in the dark.
44. During incubation, make 6.5 mL 1× Perm Buffer per sample by diluting 1 part 10× Perm Buffer with 9 parts Milli-Q water.
45. After incubation, add 2 mL 1× Perm Buffer and spin down cells for 5 min at 485 g.
46. Decant, and add another 2 mL 1× Perm Buffer.
47. Wash cells again via centrifugation.
48. During second spin, make intracellular antibody master mix in 100 μL 1× Perm Buffer containing titrated amounts of the following antibodies:
  a. FoxP3-PE/Dazzle 594 (206D).
  b. CTLA-4-BV786 (BNI3).
  c. Helios-PE/Cy7 (22F6).
  d. HLA-B7 Tetramer-PE.
49. Add antibody master mix to cells.
50. Vortex cells, and incubate at room temperature for 30 min.
51. Wash cells with 2 mL 1× Perm Buffer, spin for 5 min at 485 g.
52. Wash cells again with 3 mL flow buffer, spin for 5 min at 485 g.
53. Resuspend cells in 200 μL flow buffer for analysis on flow cytometer.

### LAP assay

**Timing: 2 h**

**Figure 7:**
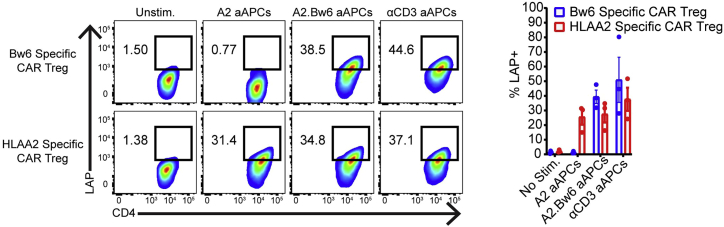
Example data for LAP assay. Bar graph represents mean ± SEM of three independent experiments.

**Figure 8:**
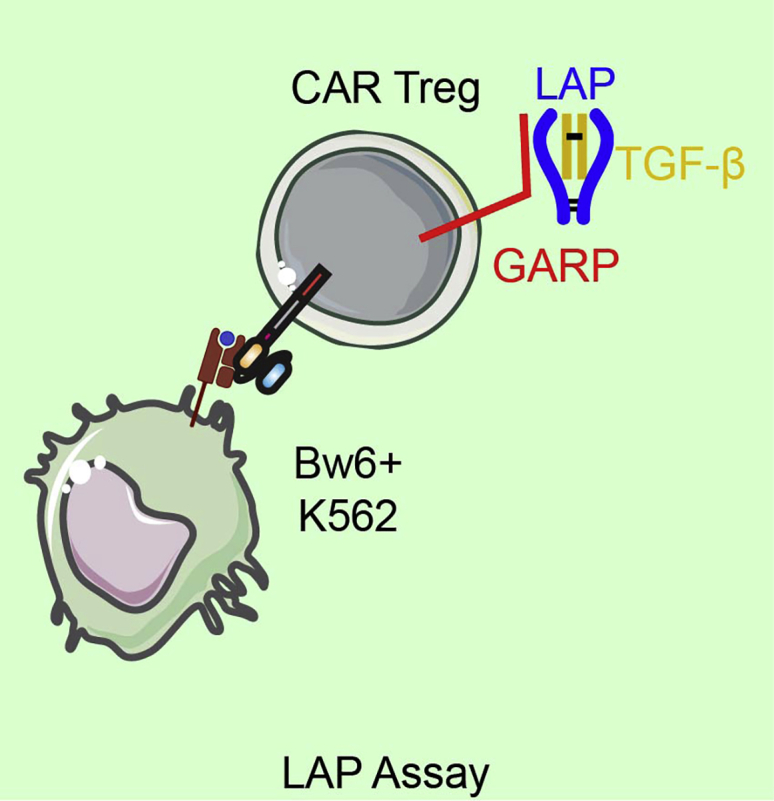
LAP assay After 24 h of co-culture between CAR Tregs and K562s, Tregs are stained for expression of LAP to determine antigen specificity of CAR Tregs.

The following protocol allows the researcher to determine the extent of antigen specific activation of the CAR Treg product by measuring cell surface expression of the TGFβ propeptide LAP. While the assay using aAPC targets bearing the antigen of interest is described, this assay has also been successful with plate-bound antigen. Example of expected results can be found in Figure 7. A graphical representation of the LAP assay is depicted in Figure 8.

54. Thaw 1 vial each of Bw6- K562 cells (here K562.HLA-A2 cells are used), Bw6+ K562 cells (here we used K562.HLA-A2.Bw6 cells), and K562.F12Q.86 cells as above.
55. Resuspend cells at 4 × 10^6^ cells/mL in R10.
56. Wash CAR Tregs with R10 and resuspend cells at 2 × 10^6^ cells/mL.
57. Add 50 μL (0.1 × 10^6^ cells) CAR Tregs to each of 4 wells in a 96 well polystyrene round bottom plate and then add one of the following:
  a. 50 μL R10 medium (no stimulation).
  b. 50 μL parental K562 (0.2 × 10^6^ cells, off target).
  c. 50 μL K562.Bw6 cells (on target).
  d. 50 μL K562.F12Q.86 (positive control stimulus).
58. Add 100 μL of R10 containing 600 IU/mL IL-2 to each well.
59. Incubate cells for 24 h at 37°C/5% CO_2_.
60. The following day, put entire contents of each well into a flow cytometry tube containing 3 mL flow buffer.
61. Spin down cells at 485 g for 5 min.
62. During spin, resuspend the titrated amounts of the following reagents in 100 μL flow cytometry buffer per sample:
  a. CD4-BV421 (OKT4).
  b. CD8α-BV510 (RPA-T8).
  c. LAP-APC (TW4-6H10).
  d. Fixable Viability Dye eFluor 780.
63. Add 100 μL of the above master mix to each sample.
64. Incubate cells for 15 min at 4°C.
65. Wash tubes with 3 mL flow cytometry buffer, spin down at 485 g for 5 min.
66. Resuspend cells in 200 μL of 2% PFA and analyze on flow cytometer.

### Loading PBMCs with CellTrace Violet (CTV) for use in Treg suppression assay

**Timing: 30 min**

Batches of PBMCs need to be loaded and frozen for use in Treg suppression assays. As the amount of CTV used can impact proliferation and viability of PBMCs, we have described our method below. The CTV labeled before freezing is well maintained, and doing so in larger batches increases consistency between experiments and saves time.

67. Obtain PBMCs from NHP blood draw as described in the method “isolation of PBMCs from Cynomolgus macaque whole blood”.
68. Remove 1 vial of CTV from freezer.
69. Allow vial to warm to room temperature before opening.
70. Pulse spin tube to pool reagent at bottom of tube.
71. Resuspend in 20 μL anhydrous DMSO to generate 5 mM stock solution.
72. Wash cells once with PBS and resuspend up to 25 × 10^6^ PBMCs in 1 mL PBS.
73. Dilute CTV stock 1:100 to 50 μM.
74. Add 110 μL of 50 μM working stock for every 1 mL of cells in PBS for a final concentration of 5 μM.
75. Mix well and incubate for 20 min at room temperature.

***Note:*** CellTrace proliferation dyes will undergo hydrolysis in aqueous solutions to become non-cell permeable. Dilute stock with PBS just before labeling cells.

76. To remove free dye from solution, add 5 volumes of R10 and incubate at room temperature for 5 min.
77. Spin cells down for 5 min at 485 g and freeze in aliquots of 2.5 × 10^6^ cells/vial in 90% FBS / 10% DMSO.

### Treg suppression assay

**Timing: 4 days**

**Figure 9:**
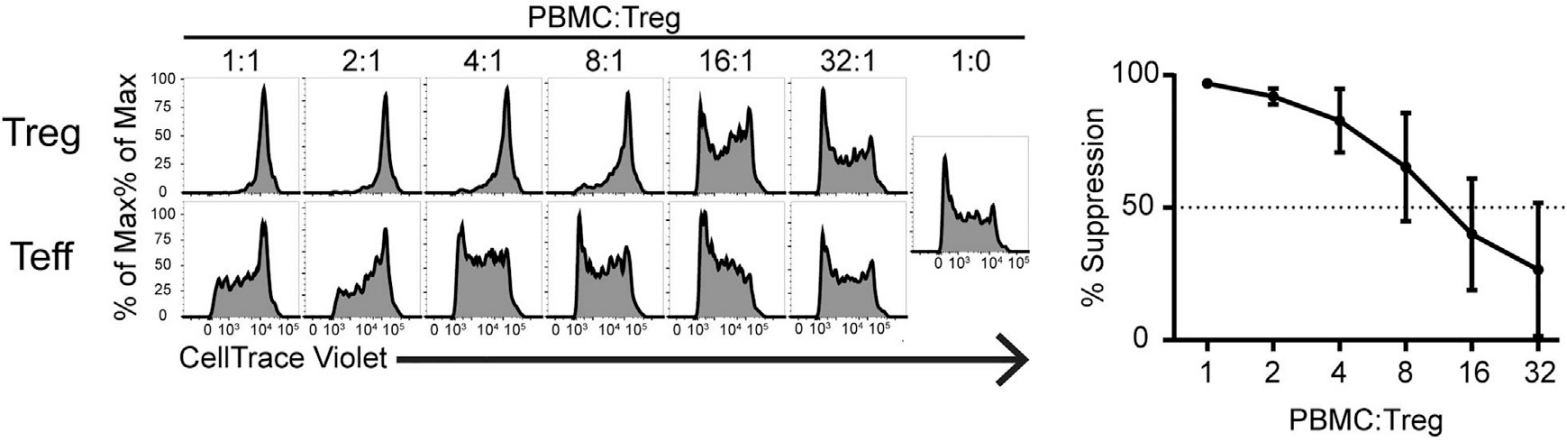
Example data for Treg suppression assay. Line graph represents mean ± SEM of seven independent experiments.

**Figure 10:**
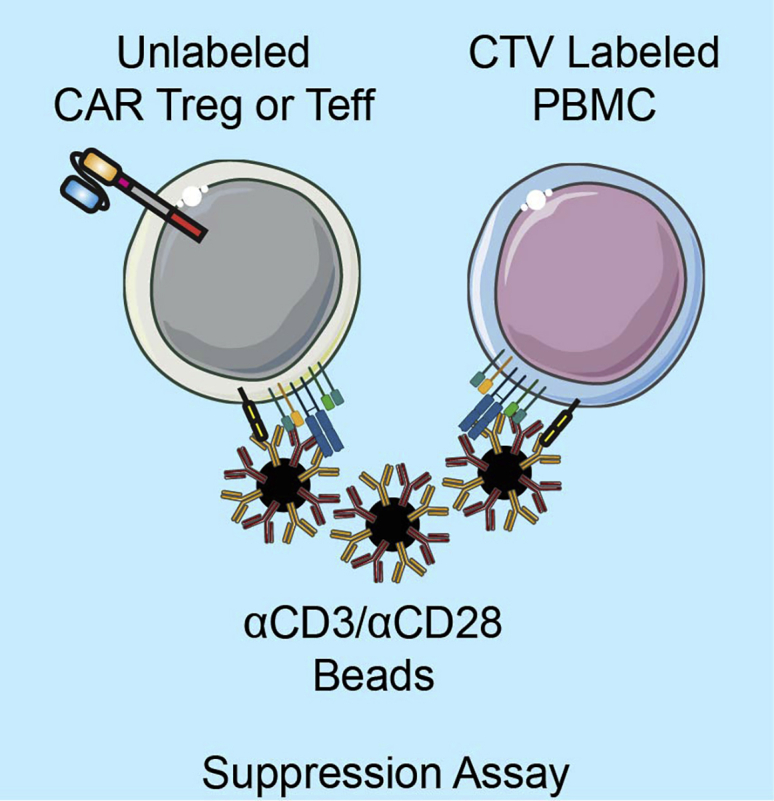
Treg suppression assay Measurement of CTV dilution after co-culture between Tregs, CTV labeled PBMCs, and α-CD3/α-CD28 beads to assess Treg suppressor ability.

This assay quantifies the antigen non-specific suppressive activity of CAR Tregs. We suggest dedicating a large quantity of PBMCs that have been labeled with cell proliferation dye and frozen to standardize responders across batches of CAR Tregs. We find that irradiated aAPCs are too strong of a stimulus for Treg suppression assays, so the T cell Activation/Expansion Kit (Miltenyi Biotec) is used instead. See Table 1 for an example of the plate setup. Example data are depicted in Figure 9, and a graphical representation of the suppression assay can be found in Figure 10.

78. Wash CAR Tregs and Teffs with R10 medium three times to eliminate residual IL-2.
79. Resuspend cells in R10 at 750k cells/mL.
80. Thaw frozen, CTV labeled PBMCs as above.
81. Wash cells twice with R10 medium and resuspend at 750k cells/mL in R10.
82. Wash α-CD3/α-CD28 beads generated in the section “loading of T cell stimulation particles with α-CD3 and α-CD28 for Treg suppression assay”.
  a. Add 20 μL of α-CD3/α-CD28 beads to 100 μL of R10 in an Eppendorf tube.
  b. Spin cells in a tabletop centrifuge for 5 min at 300 × *g*.
  c. Remove media, resuspend beads in 500 μL R10 medium, and count. Adjust beads to 375k beads/mL.
83. Add 134 μL Tregs (100k cells) to well B2 in a 96 well round bottom polystyrene plate.
84. Fill up wells B3-B7 with 67 μL fresh R10.
85. Perform 1:1 serial dilution of Tregs by moving 67 μL of cells from B2 through B7.
86. Remove the last 67 μL from well B7 so that all wells finish this step containing 67 μL of CAR Tregs.
87. Add 67 μL of CTV labeled PBMCs (50k cells) to each well B2-B7.
88. Add 67 μL of beads (25k beads) to each well B2-B7.
89. Repeat steps 83–88 with Teffs instead of Tregs in wells C2-C7.
90. Create a row of control wells:
  a. Add 67 μL of PBMCs and 134 μL R10 to well D2 as an unstimulated control.
  b. Add 67 μL of PBMCs, 67 μL beads, and 67 μL R10 to well D3 as an unsuppressed control.
  c. Add 67 μL PBMCs, 67 μL Tregs, and 67 μL R10 to wells D4-D7 to use as a single color CTV compensation control for flow cytometry.
91. Fill surrounding wells with 200 μL PBS.
92. Put plate in 37°C/5% CO_2_ incubator for 4 days.
93. Stain each well for flow cytometry analysis as described above with titrated amounts of the following reagents in 100 μL flow buffer:
  a. CD4-AF488 (OKT4).
  b. CD8α-BV510 (RPA-T8).
  c. Fixable Viability Dye eFluor 780.

***Note:*** To accurately compensate CTV fluorescence, pool wells D4-D7 and leave unstained. These cells can also be used to set the proper voltage for your flow cytometer.

94. Use FlowJo’s proliferation platform to determine division index for each tube. This represents the average number of divisions per cell in each sample.
95. To determine level of suppression, compare division indices of suppressed and unsuppressed using the following formula:

**Table 1:** Setup of suppression assay

2	3	4	5	6	7
**B**					

50,000 CAR Tregs	25,000 CAR Tregs	12,500 CAR Tregs	6,250 CAR Tregs	3,125 CAR Tregs	1,562 CAR Tregs
50,000 CTV PBMCs	50,000 CTV PBMCs	50,000 CTV PBMCs	50,000 CTV PBMCs	50,000 CTV PBMCs	50,000 CTV PBMCs
25,000 Beads	25,000 Beads	25000 Beads	25,000 Beads	25,000 Beads	25,000 Beads

**C**					

50,000 CAR Teffs	25,000 CAR Teffs	12,500 CAR Teffs	6,250 CAR Teffs	3,125 CAR Teffs	1,562 CAR Teffs
50,000 CTV PBMCs	50,000 CTV PBMCs	50,000 CTV PBMCs	50,000 CTV PBMCs	50,000 CTV PBMCs	50,000 CTV PBMCs
25,000 Beads	25,000 Beads	25,000 Beads	25,000 Beads	25,000 Beads	25,000 Beads

**D**					

		50,000 CAR Tregs	50,000 CAR Tregs	50,000 CAR Tregs	50,000 CAR Tregs
50,000 CTV PBMCs	50,000 CTV PBMCs	50,000 CTV PBMCs	50,000 CTV PBMCs	50,000 CTV PBMCs	50,000 CTV PBMCs
	25,000 Beads

$\text{\% suppression }=\left(1-\frac{Division\;index_{Suppressed}}{Division\;index_{Unsuppressed}}\right)*100$

## Expected outcomes

From an initial population of 25k–100k sorted Tregs, we expected between 500–1000 × 10^6^ cells on day 18–21 ready for freezing (Figure 6A). By flow, our final cell product is expected to be >80% FoxP3+, >80% Helios+, and >80% CAR+. Levels of CTLA-4 were variable in this protocol (Figures 6B and 6C). Each batch of Tregs was subject to LAP and suppression assays, where we expected >30% LAP+ in response to target antigen (Figure 7), and 50% suppression occurring between 8:1 and 16:1 PBMC: Treg ratio (Figure 9). Depending on the strength of bead stimulus in the suppression assay and the composition of PBMCs used, the suppressor ability of the Tregs may be less or more apparent. Comparison to Teff cells as the suppressors is therefore vital to interpretation of the experiment.

## Limitations

The ability to generate large batches of Tregs is dependent on the ability to get high levels of transduction during the first week of growth. Low transduction abilities stifles growth during week 2, affecting overall cell numbers. In rare occasions, Tregs were found to have low suppressor ability *in vitro*, which correlated with lower percentage of FoxP3+ cells, and were thus discarded. We believe that these batches were due to non-stringency during the sorting step. Suppressive batches were later able to be generated from the same animals by using more stringent CD25+ gating. For animal health, IACUC limits both the volume and frequency of blood draws.

## Troubleshooting

### Problem 1

Transduction percentage is low (step 24).

### Potential solution

In the case where transduction percentage is low, increasing the amount of virus may help increase the transduction percentage. Alternatively, the batch of virus may not have been optimally generated. In this case, first ensure that HEK 293T cells are healthy and grow to 70%–90% confluency every 2 days. If not, replace unhealthy HEK 293T cells with another frozen batch. Next, check the fidelity of the transfer plasmid, which should have been isolated with an endotoxin free maxiprep kit. When run on an agarose gel, DNA should be supercoiled and free of genomic DNA contamination. Liberating the CAR insert with restriction enzyme digestion should generate DNA fragments of the correct size. Re-sequence the DNA transgene by Sanger sequencing, as viral transfer plasmids are prone to recombination due to homology in long terminal repeats.

### Problem 2

Treg expansion is poor (expansion and transduction of CAR Tregs).

### Potential solution

Check that the aAPCs still express both CD86 and αCD3 by flow cytometry by staining with His-Tagged Cynomolgus CD3ε protein, followed by CD86-BV421 and α-His Tag-AF647. As stimulation requires T cell–aAPC contact, plating in cell culture plates with too large of a surface area may prevent activation. Err on using a plate with a smaller surface area. Ensure that IL-2 was thawed no more than 2 weeks prior. Poor initial stimulation could also be due to cell death during the sorting process. Ensure that a low-pressure sort is used and that the cells are sorted into polypropylene tubes cushioned with cell culture media.

### Problem 3

Tregs are not suppressive and/or do not express FoxP3 (step 21).

### Potential solution

Stringent sorting of Tregs is essential to maintaining FoxP3 and suppressor ability in expanded cells. If cells are not phenotypically or functionally Tregs at the end of culture, re-assess sorting gates by moving CD25 and or CD45RA gate towards higher expression. Sorted Tregs should be in the top 1%–2% of CD25+ cells among CD4+ CD8- cells.

### Problem 4

PBMC yield is low following isolation from peripheral blood (step 8).

### Potential solution

As the isolation of PBMCs from *Cynomolgus macaque* blood is more difficult than from human blood, yield may be low. Typically, we recover between 50 × 10^6^–100 × 10^6^ PBMCs from 15–20 mL of blood. If recovery is low, try pipetting more of the hazy middle layer in step 8, including some of the red blood cell layer, to ensure that all PBMCs are removed from the tube. These excess red blood cells will be efficiently lysed in step 11. Additionally, perform a complete blood count on your NHP to check for lymphopenia.

### Problem 5

Treg suppression assay is not optimal (Treg suppression assay).

### Potential solution

If the Tregs have high expression of FoxP3, CD25, and Helios and stimulation-induced LAP activation but are not suppressive in the suppression assay, you may need to optimize the assay. Excess activation of PBMCs can render the Tregs unable to provide any noticeable suppression. Ensure that all IL-2 has been washed out of the assay by washing Tregs three times before use, and that the amount of α-CD3/α-CD28 beads in each well is accurate by carefully counting the beads. If there is no/low proliferation in any wells including the unsuppressed condition, try lowering the amount of CTV loaded into the PBMCs, increasing number of α-CD3/α-CD28 beads, or increasing the duration of the experiment. Additionally, check the viability of the CTV labeled PBMCs after thawing, as warming too slowly or freezing too quickly can have drastic effects.

## Resource availability

### Lead contact

Further information and requests for resources and reagents should be directed to and will be fulfilled by the lead contact, James Riley (rileyj@upenn.edu).

### Materials availability

aAPCs expressing pan-primate αCD3 and human CD86 are available from the lead contact with a completed Materials Transfer Agreement.

## Data Availability

This protocol did not develop any code.
